# Association Between Urinary Phthalate Metabolites and Early Spontaneous Abortion

**DOI:** 10.3390/toxics14040300

**Published:** 2026-03-30

**Authors:** Lin Tao, Nian Wu, Lulu Dai, Shimin Xiong, Dengqing Liao, Yuanzhong Zhou, Xubo Shen

**Affiliations:** 1 School of Public Health, Zunyi Medical University, Zunyi 563000, China; cdctaolin@163.com (L.T.);; 2 Key Laboratory of Maternal and Child Health and Exposure Science, Guizhou Provincial Department of Education, Zunyi 563000, China; 3 Centre for Disease Control and Prevention, Huaxi District, Guiyang 550025, China

**Keywords:** phthalates, spontaneous abortion, early pregnancy

## Abstract

Phthalates (PAEs) are ubiquitous endocrine-disrupting chemicals (EDCs), but their association with early pregnancy loss (gestational age ≤ 12 weeks) remains controversial. This study enrolled pregnant women aged 20–45 years in Zunyi City, China, and included 107 cases and 349 controls following propensity score matching. Logistic regression, restricted cubic spline (RCS) analysis, Bayesian kernel machine regression (BKMR), and weighted quantile sum (WQS) regression were employed to investigate associations between urinary phthalate metabolites and early pregnancy loss. We found that monoethyl phthalate (MEP), mono(2-ethylhexyl) phthalate (MEHP), monooctyl phthalate (MOP), mono(2-ethyl-5-oxohexyl) phthalate (MEOHP), and mono(2-ethyl-5-hydroxyhexyl) phthalate (MEHHP) were associated with spontaneous abortion in early pregnancy, with corresponding odds ratios (ORs; 95% confidence intervals [CIs]) of 1.62 (1.26–2.09), 1.49 (1.07–2.09), 1.64 (1.26–2.12), 1.78 (1.27–2.50), 2.63 (1.90–3.64), 1.41 (1.11–1.79), and 5.39 (3.53–8.25). Non-linear dose–response relationships were observed between exposure to MMP, MEP, MEHP, MOP, monobenzyl phthalate (MBZP), MEOHP, MEHHP, and mono-(3-carboxypropyl) phthalate (MECPP) and early pregnancy loss (non-linear *p* < 0.05; overall *p* < 0.05). Co-exposure to multiple phthalate metabolites was also linked to a significantly non-linear elevation in the risk of early pregnancy loss (OR; 95% confidence interval [CI]) of 1.92 (1.76–2.15). Among these metabolites, MMP, MOP, MEOHP, and MECPP make the largest contribution to the correlation. In summary, our findings indicate that exposure to phthalate esters during early pregnancy is associated with early pregnancy loss, with MMP, MOP, MEOHP, and MECPP as the primary contributors. However, these results are based on a single urine sample, and caution is warranted when interpreting the findings.

## 1. Introduction

Phthalates (PAEs), widely used as plasticizers and solvents, are environmental endocrine disruptors with well-documented human exposure pathways, including indoor air and household dust [1,2]. Produced annually at a scale exceeding 18 billion pounds, these chemicals contaminate environmental matrices, wildlife, and human tissues; their metabolites have been detected in maternal blood, cord blood, placental tissue, and amniotic fluid [3,4]. Parent PAEs are metabolized in the gastrointestinal tract and saliva to monoester metabolites, which—rather than the parent compounds themselves—mediate toxicological effects, including disruption of normal biological functions following sustained low-level exposure [5,6,7].

Spontaneous abortion (SA), defined as pregnancy loss before 28 weeks of gestation and subclassified into early (≤12 weeks) and late (12–28 weeks) types [8,9], has shown conflicting associations with prenatal PAE exposure: some studies suggest that PAE metabolites are associated with spontaneous abortion [10,11], while others find no link [12,13]. In environmental epidemiology, human PAE exposure is almost always mixed rather than to individual compounds. Single-pollutant models are ill-suited to investigating the PAE–SA association, as they fail to capture nuanced exposure–outcome interactions. These models only quantify the effects of individual PAE metabolites, missing synergistic, antagonistic, or interactive effects of co-occurring PAEs—ubiquitous endocrine disruptors whose combined exposure may induce additive toxicity, risking underestimation or misclassification of overall risk. They also suffer from multicollinearity in multi-pollutant datasets, biasing regression estimates, reducing statistical power, and contributing to study inconsistencies; additionally, they typically assume linear exposure–response relationships, overlooking non-linear associations and low-dose mixed exposure risks.

To address these limitations, we used two complementary mixture models: weighted quantile sum (WQS) regression and Bayesian kernel machine regression (BKMR). The WQS model mitigates multicollinearity, quantifies mixed exposure effects, and identifies key contributing metabolites [14,15]. BKMR, an advanced Bayesian semi-parametric approach, flexibly models non-linear relationships and pollutant interactions without prespecifying effect directions—mimicking real-world exposure scenarios—with visualization tools enhancing conclusion robustness. Together, these frameworks overcome the limitations of single-pollutant models, enabling rigorous assessment of the mixed PAE exposure–SA association and providing robust statistical support.

Here, we investigate the association between urinary PAE metabolites and early SA (≤12 weeks) in pregnant women from Zunyi City, aiming to provide insights for maternal–fetal health protection in western China.

## 2. Materials and Methods

### 2.1. Zunyi Birth Cohort and Study Population

**Figure 1:**
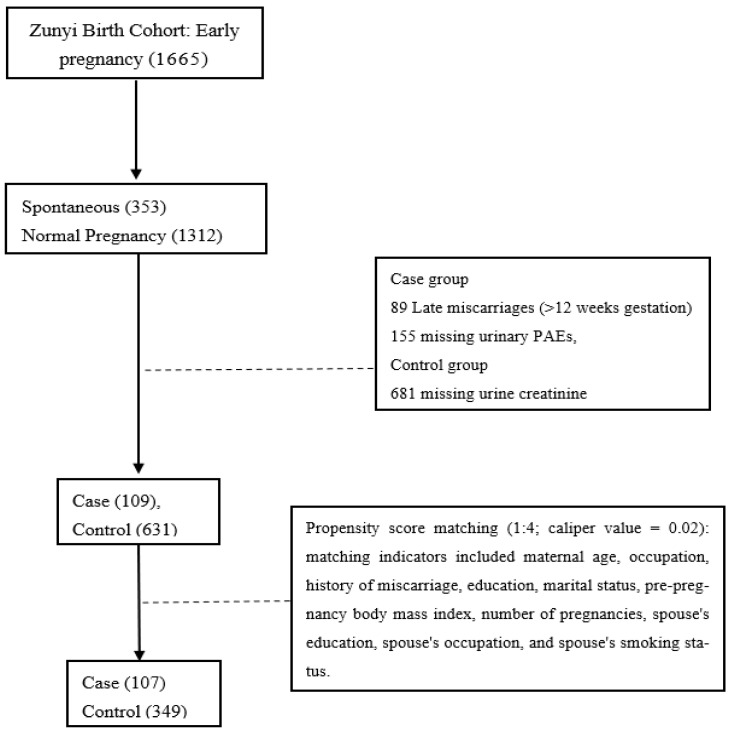
Study population.

The Zunyi Birth Cohort (ZBC, *n* = 6583) is a prospective study established between April 2020 and May 2022, investigating the impacts of environmental and behavioral factors on fetal development and pregnancy outcomes. Details of the cohort have been previously described [16,17]. This study employed a case–control design. The case group comprised women with ultrasound-confirmed early spontaneous abortion (gestation ≤ 12 weeks), excluding pregnancy loss due to induced termination, ectopic pregnancy, hydatidiform mole, or other pathological causes. The control group consisted of women with intrauterine pregnancies that progressed to full-term live birth, with no history of spontaneous abortion, threatened abortion, or adverse pregnancy outcomes. Initially, 1665 pregnant women in early pregnancy were enrolled, comprising 353 cases and 1312 controls. Subsequent exclusions included 89 cases with mid-to-late pregnancy miscarriages, 155 cases, and 681 controls lacking urinary PAE metabolite data. To mitigate data bias and confounding variables, propensity score matching (1:4 ratio, caliper value = 0.02) was applied, matching on maternal age, occupation, miscarriage history, education, marital status, pre-pregnancy BMI, parity, spouse’s education, spouse’s occupation, and spouse’s smoking status. The final matched sample included 107 cases and 349 controls with a matching rate of 98.2% (Figure 1). The study was approved by the Ethics Committee of Zunyi Medical College (No. [2019] H-005).

### 2.2. Propensity Score Matching and Variable Selection

To mitigate data bias and confounding effects, we employed propensity score matching (PSM), a statistical technique for observational studies that balances baseline characteristics to enable valid experimental–control group comparisons. Guided by a *The Lancet* study identifying miscarriage risk factors—maternal age, BMI, prior miscarriage, smoking, alcohol use, stress, occupation, air pollution, and pesticide exposure [18]—our matched variables included maternal age, occupation, miscarriage history, education, marital status, pre-pregnancy BMI, parity, spouse’s education, spouse’s occupation, and spouse’s smoking status.

### 2.3. Urine Collection and Testing

Urine sample collection and PAE metabolite detection were conducted in accordance with the national standard ‘Determination of Semi-Volatile Organic Compounds in Environmental Samples by Gas Chromatography-Mass Spectrometry’ (HJ 834-2017) [19], ensuring standardization and reproducibility throughout the process. All urine samples were collected from subjects within the first 12 weeks of pregnancy, with case group samples obtained prior to pregnancy loss. Trained nurses provided subjects with urine collection tubes and cups; where immediate sample provision was unfeasible, collection occurred the following morning. Samples were aliquoted into four portions and stored frozen at −80 °C until analysis. For PAE metabolite analysis, samples were thawed from −80 °C water bath to 37 °C. A 1.5 mL aliquot was transferred to a test tube, to which 20 µL of deuterated internal standard solution containing MEHP-C4 and MEHHP-C4 (Cambridge, MA, USA) was added. Samples were extracted three times with n-hexane: diethyl ether (1:4 *v*/*v*), concentrated under high-purity nitrogen, and incubated with 20 µL β-glucuronidase/sulfatase (Sigma-Aldrich, Milan, Italy) at 95 °C for 45 min. For every 100 urine samples analyzed, 10% were subjected to duplicate analysis for quality control (QC). Final detection and quantification were performed using a gas chromatography–mass spectrometry system (GC-MS, Agilent 9000-7000D, Santa Clara, CA, USA), measuring mass-to-charge ratio (*m*/*z*), retention time, and peak area. Standard curves for PAE metabolites in urine were established via linear regression (see Table S1). Metabolite concentrations below the limit of detection (LOD) were estimated as LOD/√2. The LOD range for PAE metabolites was 0.0015–23.4375 μg/L, with recovery rates of 80.45–124.28%. Concurrently, urinary creatinine levels were measured using an automated biochemical analyzer (AU680) at the First Affiliated Hospital of Zunyi Medical University laboratory. Urinary phthalate concentrations (μg/L) were normalized by dividing by creatinine levels (g/L), ultimately expressed as creatinine microgram concentrations (μg/L Cr). Detailed analytical protocols are referenced in prior studies [16,17].

Key experimental phases and timelines: (1) March 2022 to May 2023: Urine collection, aliquoting, and storage at −80 °C; (2) June to August 2023: Sample thawing, extraction, and enzymatic incubation; (3) September to November 2023: GC-MS detection of PAE metabolites and creatinine analysis; (4) December 2023: Data calibration, sub-detection limit interpolation, and standardization of PAE concentrations relative to creatinine levels.

### 2.4. Quality Control

The standard addition method was employed: three spiked concentrations (low, medium, high) were added to 1.5 mL urine samples (8 replicates per concentration) to calculate recovery and precision [17]. Detection limits (LODs) were defined at a signal-to-noise ratio (S/N) of 3. For every 20 urine samples, at least one experimental blank was included, with parallel sample relative deviations ≤20% to account for background from reagents and procedures. Internal standard intensity was monitored in each run, requiring response values within ±30% of the calibration curve to mitigate instrument bias or interference. Details on LOD, limit of quantification (LOQ), recoveries, and precision for PAE metabolites are provided in Table S2 (see Table S2). The assay employed an automated chemical method (Jaffe reaction), utilizing key reagents including an alkaline buffer solution (containing 0.5–0.8 mol/L sodium hydroxide or potassium hydroxide to establish an alkaline reaction environment), picric acid solution (containing 0.04–0.06 mol/L saturated picric acid [2,4,6-trinitrophenol]), diluent (saline or 0.9% sodium chloride solution, used to dilute urine samples 1:50–1:200), and calibration standards (containing known concentrations of creatinine reference material (purity ≥ 99.0%)), with a simulated urine matrix (containing small amounts of urea and sodium chloride to approximate the actual sample matrix).

### 2.5. Statistical Methods

Outliers were excluded; missing values for age and pre-pregnancy BMI (accounting for less than 0.5%) were imputed using the mean; missing phthalate concentration values (accounting for less than 0.5%) were replaced with the median. PAE concentrations underwent log transformation prior to Kolmogorov–Smirnov testing. Urinary PAE metabolite differences between case and control groups were evaluated via Mann–Whitney U tests, while baseline maternal characteristics were compared using chi-square tests and independent samples *t*-test. We used univariate and multivariate logistic regression analyses to examine the association between the overall concentration and quartile (Q1–Q4) concentrations of PAE metabolites and spontaneous abortion. Dose–response relationships were explored using restricted cubic spline (RCS) plots, and the combined effects of PAE metabolites on spontaneous abortion were assessed via Bayesian kernel machine regression (BKMR); weighted quantile sum (WQS) regression was used to assess the weighting of PAE metabolites on spontaneous abortion. Analyses were conducted using SPSS 29.0 (IBM Corp., Armonk, NY, USA) and R v3.4.0 (The Comprehensive R Archive Network, http://cran.r-project.org, accessed on 19 March 2026).

## 3. Result

### 3.1. Baseline Data on Pregnant Women

This study enrolled pregnant women aged 20 to 45 years in Zunyi, Guizhou Province, China, at 12 weeks gestation or earlier, ultimately including 107 cases and 349 controls. Detailed information indicates that participants were predominantly aged 20 to 35 years (92%), with an unemployment rate below 20% and 80% having completed secondary or high school education. In both pre- and post-matching control groups, 30% of pregnant women had a history of spontaneous abortion. Although nearly all participants were non-smokers, both groups reported frequent exposure to second-hand smoke. Pre-matching revealed significant differences between the case and control groups in gestational age and literacy levels; these differences disappeared post-matching, indicating enhanced homogeneity within the study population (Table 1).

### 3.2. Concentrations of PAE Metabolites in Urine

The study included 456 participants, comprising 348 controls (76.3%) and 107 cases (23.7%). Urinary phthalate (PAE) metabolite concentrations were non-normally distributed and are therefore presented as medians (interquartile ranges, IQRs). Overall, monobutyl phthalate (MBP) was the most abundant urinary PAE metabolite [77.73 (35.55, 161.70) µg/L creatinine], while monobenzyl phthalate (MBZP) was the least abundant [0.06 (0.03, 0.21) µg/L creatinine]. In controls, MBP concentration was 77.73 (35.55, 166.48) µg/L creatinine and MBZP was 0.06 (0.02, 0.20) µg/L creatinine. In cases, MBP and MBZP concentrations were 77.31 (35.64, 154.75) µg/L creatinine and 0.07 (0.03, 0.28) µg/L creatinine, respectively. Mann–Whitney tests revealed significantly higher concentrations of MMP, MEHP, MOP, MEHHP, and MECPP in cases than in controls (*p* < 0.005), whereas no significant differences were observed for MEP, MIBP, MBP, MBZP, or MEOHP (*p* > 0.05; Table 2).

### 3.3. Association Between PAE Metabolites and Spontaneous Abortion

We used univariate and multivariate logistic regression models to examine the association between PAE metabolites and spontaneous abortion. Univariate analysis identified associations between spontaneous abortion and MMP, MEP, MEHP, MOP, MEOHP, MEHHP, and MECPP, with corresponding odds ratios (ORs; 95% confidence intervals [CIs]) of 1.62 (1.26–2.09), 1.49 (1.07–2.09), 1.64 (1.26–2.12), 1.78 (1.27–2.50), 2.63 (1.90–3.64), 1.41 (1.11–1.79), and 5.39 (3.53–8.25), respectively. In multivariate analysis, associations persisted for MMP, MOP, MEOHP, and MECPP, with ORs (95% CIs) of 1.54 (1.06–2.24), 2.01 (1.12–3.58), 2.86 (1.92–4.27), and 5.20 (3.22–8.41), respectively (Table 3). Further quartile-based analyses (referenced to the lowest quartile, Q1) revealed additional associations. In univariate analysis, MMP Q2–Q4 were associated with spontaneous abortion (ORs [95% CIs]: 4.42 [2.06–9.48], 5.62 [2.64–11.95], 2.77 [1.26–6.11]); MEP Q2 was associated with spontaneous abortion (OR [95% CI]: 2.08 [1.11–3.89]); MEHP Q2 and Q4 showed associations (ORs [95% CIs]: 1.93 [1.01–3.71], 2.74 [1.45–5.16]); MOP Q2 and Q4 were linked to higher risk (ORs [95% CIs]: 2.04 [1.05–3.95], 2.97 [1.56–5.65]); MEOHP Q2–Q4 were strongly associated (ORs [95% CIs]: 6.58 [2.19–19.77], 16.65 [5.73–48.42], 14.30 [4.90–41.69]); and MECPP Q4 was associated with spontaneous abortion (OR [95% CI]: 7.24 [3.65–14.36]). In multivariate analysis (referenced to Q1), MMP Q2 and Q3 were associated with spontaneous abortion (ORs [95% CIs]: 6.20 [2.39–16.08], 8.87 [3.37–23.30]); MOP Q4 showed an association (OR [95% CI]: 6.02 [1.98–18.28]); MEOHP Q2–Q4 were strongly associated (ORs [95% CIs]: 15.72 [4.41–56.05], 48.77 [13.30–178.87], 49.74 [13.14–188.26]); and MECPP Q4 was linked to spontaneous abortion (OR [95% CI]: 12.26 [4.90–30.66]) (Table 4). We also analyzed the association between PAE metabolites before creatinine correction and spontaneous abortion (see Tables S3 and S4).

### 3.4. Exposure–Dose Relationship Between PAE Metabolites and Spontaneous Abortion

**Figure 2:**
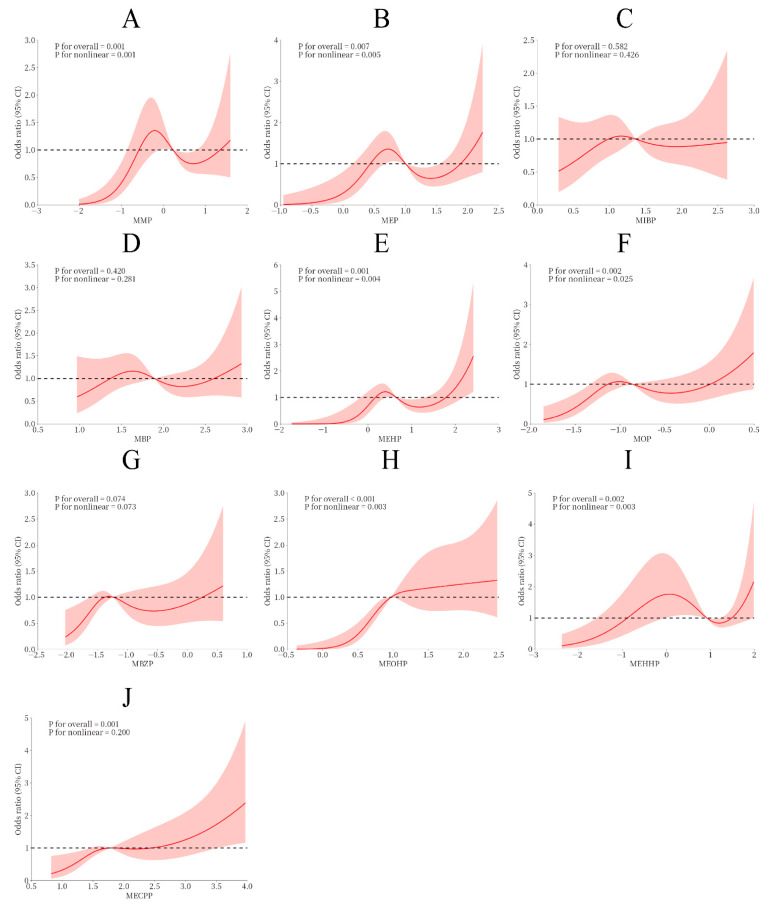
Non-linear relationship between PAEs and spontaneous abortion. The red line in the graph represents the nonlinear relationship between PAE metabolites and spontaneous abortion, the shaded part represents the 95% confidence interval, and the dotted line represents the or value of 1. Adjusting factors: Maternal age, educational attainment, history of miscarriage, number of previous pregnancies, pre-pregnancy body mass index (BMI), occupational history, husband’s educational attainment, husband’s employment history, maternal smoking status, creatinine and husband’s smoking status. MMP (mono-methyl), MEP (mono-methyl phthalate) MIBP (mono-ethyl phthalate), MBP (mono-butyl phthalate), MOP (mono-octyl phthalate), MBZP (mono-benzyl phthalate), MEHP (mono(2-ethylhexyl) phthalate), MEOHP (mono(2-ethyl-5-oxohexyl) phthalate), MEHHP (mono(2-ethyl-5-hydroxyhexyl) phthalate), MECPP (mono-5-carboxy-2-ethylpentyl ester). (**A**) The relationship between the exposure dose of MMP and spontaneous abortion; (**B**) The relationship between MEP and the exposure dose of spontaneous abortion; (**C**) The relationship between mibp and the exposure dose of spontaneous abortion; (**D**) The relationship between MBP and the exposure dose of spontaneous abortion; (**E**) The exposure dose relationship between MEHP and spontaneous abortion; (**F**) The relationship between the exposure dose of mop and spontaneous abortion; (**G**) The relationship between mbzp and the exposure dose of spontaneous abortion; (**H**) The exposure dose relationship between meohp and spontaneous abortion; (**I**) The relationship between the exposure dose of mehhp and spontaneous abortion; (**J**) The relationship between mecpp and the exposure dose of spontaneous abortion.

We further used restricted cubic spline plots to explore the dose–response relationship between PAE metabolites and spontaneous abortion. Results showed that MMP, MEP, MEHP, MOP, MBZP, MEOHP, MEHHP, and MECPP exhibited non-linear dose–response relationships with spontaneous abortion (non-linear *p* < 0.05; overall *p* < 0.05), whereas no such associations were observed for MIBP or MBP (Figure 2).

### 3.5. Association Between Mixed Exposure to PAE Metabolites and Spontaneous Abortion

**Figure 3:**
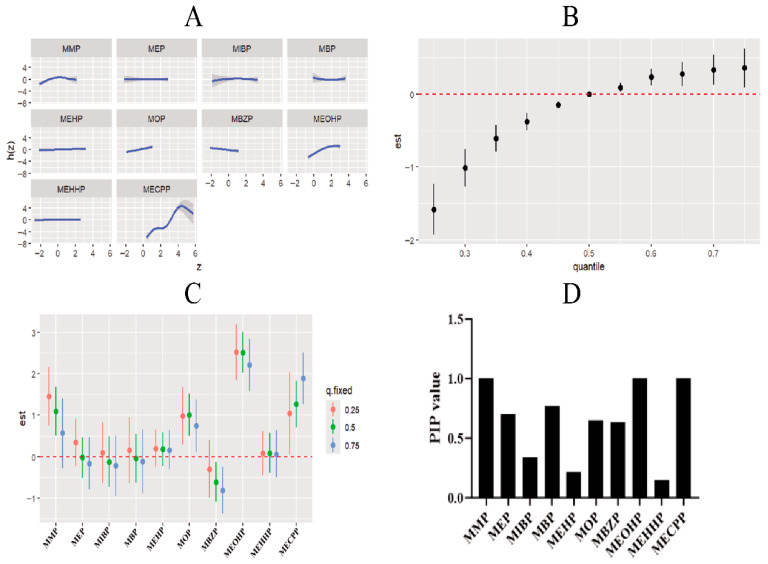
Association between mixed exposure to PAE metabolites and spontaneous abortion. (**A**) Single pollutant exposure–response function of PAE metabolites and spontaneous abortion. (**B**) Overall exposure–response function of PAE metabolites and spontaneous abortion. (**C**) Component effects of PAE metabolites and spontaneous abortion. (**D**) Posterior inclusion probability (PIP) of PAE metabolites and spontaneous abortion. Adjustment factors: Maternal age, educational attainment, history of miscarriage, number of previous pregnancies, pre-pregnancy body mass index (BMI), occupational history, husband’s educational attainment, husband’s employment history, maternal smoking status, creatinine and husband’s smoking status. MMP (mono-methyl), MEP (mono-methyl phthalate) MIBP (mono-ethyl phthalate), MBP (mono-butyl phthalate), MOP (mono-octyl phthalate), MBZP (mono-benzyl phthalate), MEHP (mono(2-ethylhexyl) phthalate), MEOHP (mono(2-ethyl-5-oxohexyl) phthalate), MEHHP (mono(2-ethyl-5-hydroxyhexyl) phthalate), MECPP (mono-5-carboxy-2-ethylpentyl ester).

We further employed Bayesian kernel machine regression (BKMR) to examine the relationship between mixed exposure to 10 phthalate (PAE) metabolites and spontaneous abortion. In single-pollutant exposure–response functions, MMP showed an inverted U-shaped relationship with spontaneous abortion, while MOP, MEOHP, and MECPP exhibited non-linear positive correlations. The overall exposure–response function revealed a pronounced non-linear positive association between combined PAE exposure and spontaneous abortion. Component effect analysis identified additional associations: positive associations between spontaneous abortion and MMP at the 25th and 50th percentiles; positive associations with MOP at the 25th, 50th, and 75th percentiles; negative associations with MBZP at the 50th and 75th percentiles; and positive associations with MEOHP and MECPP at the 25th, 50th, and 75th percentiles. Posterior inclusion probability (PIP) analysis indicated that MMP, MEOHP, and MECPP were the top three metabolites contributing most strongly to spontaneous abortion risk (Figure 3).

### 3.6. Weighted Analysis of the Association Between PAE Metabolite Exposure and Spontaneous Abortion

**Figure 4:**
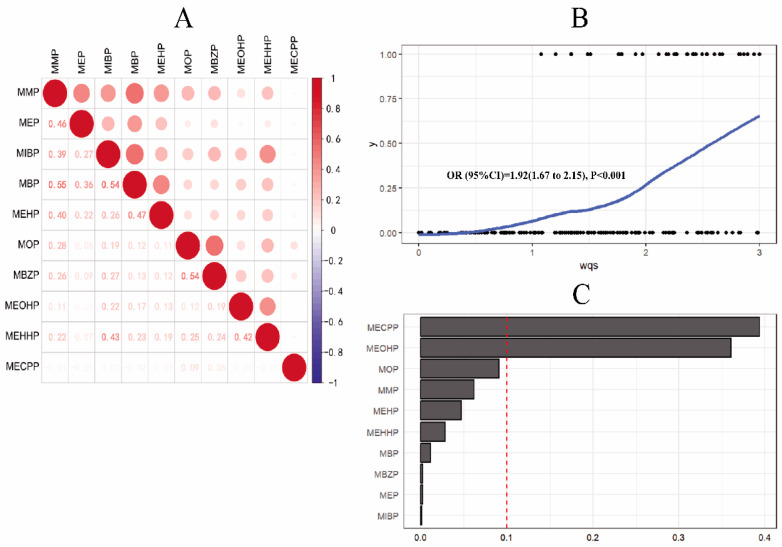
Weighted analysis of the association between PAE metabolite exposure and spontaneous abortion. factors: Maternal age, educational attainment, history of miscarriage, number of previous pregnancies, pre-pregnancy body mass index (BMI), occupational history, husband’s educational attainment, husband’s employment history, maternal smoking status, creatinine and husband’s smoking status. MMP (mono-methyl), MEP (mono-methyl phthalate) MIBP (mono-ethyl phthalate), MBP (mono-butyl phthalate), MOP (mono-octyl phthalate), MBZP (mono-benzyl phthalate), MEHP (mono(2-ethylhexyl) phthalate), MEOHP (mono(2-ethyl-5-oxohexyl) phthalate), MEHHP (mono(2-ethyl-5-hydroxyhexyl) phthalate), MECPP (mono-5-carboxy-2-ethylpentyl ester). (**A**) Correlation analysis between PAE metabolites; (**B**) The overall effect of PAE metabolites on spontaneous abortion; (**C**) Corresponding association weights of PAE metabolites in association with spontaneous abortion.

We further employed weighted quantile sum (WQS) regression to evaluate the relative contribution of each PAE metabolite to the association with spontaneous abortion. Correlation analysis revealed weak-to-moderate correlations (r < 0.6) among PAE metabolites. WQS regression showed a positive association between the PAE metabolite mixture and spontaneous abortion, with an odds ratio (OR; 95% confidence interval [CI]) of 1.92 (1.76–2.15). Weighted contribution analysis identified MMP, MOP, MEOHP, and MECPP as the top metabolites contributing most strongly to spontaneous abortion risk (Figure 4).

## 4. Discussion

PAE metabolite recoveries in this study ranged from 80.45 to 124.28%, consistent with prior reports of 60–140% [20,21]. Urinary PAE concentrations (0.06–77.73 µg/L Cr) were lower than those reported in Wuhan (0.23–112.83 µg/L Cr) and Tianjin (0.803–122.025 µg/L Cr), China [22,23], and substantially lower than in Iran (120–860 µg/L Cr) [24], potentially reflecting limited exposure sources, lower dose burdens, and diverse contaminant profiles in southwest China [25,26]. Higher urinary PAE concentrations in the case group compared to controls were observed, which is consistent with findings from other observational studies [27].

Our observation of associations between PAE metabolites and spontaneous abortion is consistent with prior observational research [28,29]. For example, Liao et al. reported associations between MEP, MIBP, DBP, and DEHP metabolites and recurrent miscarriage risk [30], while Gao et al. linked higher urinary MEP, MEOHP, and MEHHP concentrations to increased embryo loss risk [28]. Danish researchers similarly observed a positive correlation between MEHP exposure and subclinical embryo loss [31]. Our dose–response analyses further revealed distinct trends for MECPP, MEP, and MBP—findings that are rarely reported in previous studies investigating PAE exposure and spontaneous abortion. Notably, a key novelty of this study lies in our use of Bayesian kernel machine regression (BKMR) to assess the joint effects of 10 urinary PAE metabolites during early pregnancy; this approach addresses a critical gap in the field, as most prior work has focused solely on single metabolite associations rather than the combined effects of multiple PAEs.

To provide a balanced interpretation of our findings, it is important to acknowledge studies that have reported no associations or inconsistent results between PAE exposure and spontaneous abortion. For instance, a large cohort study in the United States (*n* = 1241) found no significant associations between urinary concentrations of MEP, MBP, or DEHP metabolites and spontaneous abortion risk [14]. Similarly, a case–control study in South Korea (68 cases, 136 controls) observed no statistically significant links between any PAE metabolite and early pregnancy loss [32]. These inconsistencies may stem from variations in sample size, study design, maternal characteristics, aXEbortion subtype, and sampling timing. Many studies had small sample sizes (11–150 cases) [33,34], limiting statistical power and contributing to heterogeneity. Geographic and demographic differences in PAE exposure profiles and metabolic pathways may also influence urinary concentrations [34]. Temporal variability in exposure—with some cohorts sampling pre-pregnancy urine [30,35] and case–control studies collecting post-conception samples [27,30]—adds complexity, as lifestyle factors (e.g., diet, personal care product use) may alter PAE kinetics across pregnancy stages. Additionally, differences in covariate adjustment strategies (e.g., exclusion of renal function markers or dietary factors) between studies may contribute to divergent results, highlighting the need for standardized methodologies in future research.

The potential mechanistic links between PAE exposure and spontaneous abortion primarily involve endocrine disruption, placental dysfunction, oxidative stress, and immune dysregulation, which we elaborate on below to enhance understanding of the observed associations. First, animal studies have identified potential endocrine-disrupting pathways: for example, dibutyl phthalate (DBP) suppresses progesterone secretion in rats and increases miscarriage risk [36,37], underscoring the need for large-scale, multi-cohort studies to validate these observational associations and clarify their biological underpinnings. Despite conflicting epidemiological findings on PAE metabolites and spontaneous abortion, toxicological studies link prenatal di(2-ethylhexyl) phthalate (DEHP) exposure to disrupted placental thyroid hormone receptor signaling in mice, leading to intrauterine growth restriction [38,39]—a condition closely associated with increased spontaneous abortion risk. Second, PAE metabolites generate reactive oxygen species (ROS) during metabolism; excessive ROS disrupts redox balance, induces oxidative damage, and impairs embryonic cell proliferation and differentiation, thereby potentially increasing miscarriage risk [28]. Finally, PAEs interfere with reproductive hormone metabolism, altering progesterone levels in a manner that may elevate miscarriage risk [40], and modulate immune cell function, which could enhance maternal immune rejection of the embryo [41]. Collectively, these toxicological mechanisms provide biological plausibility for the observational associations observed in our study, though direct evidence in human pregnancies remains limited.

The public health implications of our findings are noteworthy, particularly given the widespread exposure to PAEs in daily life through food packaging, personal care products, and environmental contaminants. Early pregnancy is a critical window of vulnerability, and even low-level PAE exposure may contribute to adverse pregnancy outcomes. Our results highlight the need for enhanced surveillance of PAE exposure in reproductive-age women, particularly in southwest China, where exposure profiles differ from other regions. Additionally, our findings support the development of targeted public health interventions to reduce PAE exposure, such as promoting the use of non-phthalate alternatives in consumer products, improving food safety standards, and raising awareness among women of childbearing age about potential exposure sources. Future public health strategies should also prioritize vulnerable populations, including women with a history of miscarriage, who may be more susceptible to the effects of PAE exposure.

In conclusion, our study observes associations between urinary PAE metabolites and spontaneous abortion in early pregnancy, using BKMR to highlight the joint effects of multiple PAE metabolites. These findings are consistent with some prior studies but contrast with others, underscoring the need for standardized methodologies and larger multi-cohort studies. Toxicological evidence provides biological plausibility for the observed associations, and our results have important public health implications for reducing PAE exposure in reproductive-age women. Addressing the limitations of this study will be critical for advancing our understanding of the role of PAE exposure in adverse pregnancy outcomes.

## 5. Strengths and Limitations

This study employed multiple statistical methods—logistic regression, Bayesian kernel regression, restricted cubic spline plots, and WQS regression—to assess the association between early pregnancy exposure to PAE metabolites and spontaneous abortion. However, several limitations exist: firstly, PAE metabolite levels were measured at a single time point, potentially failing to accurately reflect trends throughout the entire pregnancy. Further validation through external data and in vitro/in vivo experiments is required to substantiate these findings. Secondly, the small sample size resulted in limited statistical power, positioning this study primarily as a hypothesis-generating foundation for subsequent research. Moreover, exposure classification may be subject to error, as early pregnancy PAE exposure levels were assessed based solely on a single urine sample. PAE metabolite concentrations in urine exhibit temporal variability, and a single sample may inadequately reflect long-term exposure patterns, potentially leading to misclassification of exposure levels. Moreover, residual confounding factors cannot be entirely excluded. Although key covariates (such as age, BMI, history of miscarriage, and creatinine levels) were adjusted for, unmeasured or incompletely measured factors—including dietary patterns, exposure to other environmental pollutants (e.g., heavy metals, pesticides), and potential reproductive system disorders—may still influence the observed associations. Moreover, data on miscarriage subtypes (e.g., embryonic versus fetal loss) were not collected, and sensitivity to PAE exposure may differ across miscarriage types. Finally, this study focused on early pregnancy, and its findings may not be generalizable to later gestational periods or other adverse reproductive outcomes.

## 6. Conclusions

The study identified an association between PAE metabolites in maternal urine during the first trimester of pregnancy and spontaneous abortion, revealing potential synergistic effects and dose–response relationships, with MMP, MOP, MEOHP and MECPP contributing most significantly.

## Figures and Tables

**Table 1:** Basic information.

		Before PSM				After PSM (1:4)			
Variable		Control *n* = 631	Case *n* = 109	*F/t*	*p*	Control *n* = 349	Case *n* = 107	*F/t*	*p*
Age (years)	18–20 20–35 >35	30 567 34	3 101 5	1.029	0.598	13 317 19	3 99 5	0.316	0.854
Gestational week	—	7.71 ± 0.11	8.98 ± 0.28	23.20	<0.001	8.11 ± 0.12	8.93 ± 0.28	1.80	0.18
Occupation	no yes	220 411	29 80	2.840	0.092	117 232	29 79	1.698	0.194
Education	low mid high	23 488 120	5 71 33	7.741	0.02	11 255 83	5 71 31	1.938	0.397
Marriage	no yes other	34 591 6	7 100 2	0.888	0.642	329 17 3	99 6 2	0.876	0.645
Pre-pregnancy BMI (kg/m^2^)	<18.5 18.5–23.9 >23.9	67 401 163	12 70 27	0.061	0.970	38 220 91	11 69 27	0.078	0.962
Abortion history	yes no other	291 170 170	58 21 30	3.125	0.210	164 86 99	57 21 29	1.591	0.451
Pregnancies	once multiple	170 461	30 79	0.016	0.900	99 250	29 78	0.065	0.799
Spouse’s education	low mid high	10 497 124	2 80 27	1.573	0.455	6 266 77	2 79 26	0.253	0.881
Spouse’s occupation	no yes	89 542	18 91	0.436	0.509	59 290	19 88	0.042	0.838
Spouse’s smoking	yes no	256 375	38 71	1.265	0.261	134 215	40 69	0.102	0.750

low: Below primary school; mid: Middle school; high: College degree or above.

**Table 2:** Median levels of PAEs in urine of pregnant women (μg/L Cr).

Variables	Total (*n* = 456)	Control (*n* = 349)	Case (*n* = 107)	Statistic	*p*
MMP, M (Q_1_, Q_3_)	1.69 (0.23, 4.39)	1.44 (0.13, 4.55)	1.97 (0.79, 4.15)	Z = −2.48	0.013
MEP, M (Q_1_, Q_3_)	10.07 (3.71, 21.14)	10.12 (3.15, 20.77)	10.01 (4.61, 23.31)	Z = −1.28	0.202
MIBP, M (Q_1_, Q_3_)	22.96 (9.96, 50.80)	22.89 (9.66, 49.07)	23.64 (10.17, 52.16)	Z = −0.47	0.642
MBP, M (Q_1_, Q_3_)	77.73 (35.55, 161.70)	77.73 (35.55, 166.48)	77.31 (35.64, 154.75)	Z = −0.08	0.939
MEHP, M (Q_1_, Q_3_)	4.46 (1.62, 15.43)	4.20 (1.46, 13.45)	5.33 (2.28, 19.75)	Z = −2.41	0.016
MOP, M (Q_1_, Q_3_)	0.14 (0.05, 0.38)	0.13 (0.04, 0.34)	0.17 (0.07, 0.53)	Z = −2.77	0.006
MBZP, M (Q_1_, Q_3_)	0.06 (0.03, 0.21)	0.06 (0.02, 0.20)	0.07 (0.03, 0.28)	Z = −1.61	0.108
MEOHP, M (Q_1_, Q_3_)	6.67 (2.29, 22.91)	6.68 (2.08, 25.30)	6.67 (3.64, 18.82)	Z = −0.20	0.841
MEHHP, M (Q_1_, Q_3_)	8.75 (3.70, 20.85)	8.44 (3.30, 19.94)	10.45 (5.52, 23.25)	Z = −2.21	0.027
MECPP, M (Q_1_, Q_3_)	58.75 (22.18, 263.16)	52.42 (20.94, 206.15)	85.10 (33.82, 706.48)	Z = −3.55	<0.001

Z: Mann–Whitney test. M: Median, Q_1_: 1st quartile, Q_3_: 3rd quartile. Non-parametric tests (Z-tests) were employed to analyze differences in PAE metabolite concentrations between case and control groups, with *p* < 0.05 deemed statistically significant. MMP (mono-methyl), MEP (mono-methyl phthalate) MIBP (mono-ethyl phthalate), MBP (mono-butyl phthalate), MOP (mono-octyl phthalate), MBZP (mono-benzyl phthalate), MEHP (mono(2-ethylhexyl) phthalate), MEOHP (mono(2-ethyl-5-oxohexyl) phthalate), MEHHP (mono(2-ethyl-5-hydroxyhexyl) phthalate), MECPP (mono-5-carboxy-2-ethylpentyl ester).

**Table 3:** Association between PAE metabolites and spontaneous abortion.

Variables	Univariate		Multivariate	
	OR (95%CI)	*FDR-Adjusted p-Value*	OR (95%CI)	*FDR-Adjusted p-Value*
MMP	1.62 (1.26~2.09)	<0.001	1.54 (1.06~2.24)	0.010
MEP	1.49 (1.07~2.09)	0.014	0.95 (0.55~1.62)	0.762
MIBP	1.14 (0.78~1.67)	0.438	0.70 (0.33~1.48)	0.276
MBP	1.14 (0.74~1.76)	0.543	0.51 (0.23~1.16)	0.054
MEHP	1.64 (1.26~2.12)	<0.001	1.25 (0.86~1.81)	0.168
MOP	1.78 (1.27~2.50)	<0.001	2.01 (1.12~3.58)	0.005
MBZP	1.28 (0.94~1.74)	0.097	1.40 (0.73~1.71)	0.12
MEOHP	2.63 (1.90~3.64)	<0.001	2.86 (1.92~4.27)	<0.001
MEHHP	1.41 (1.11~1.79)	0.003	1.02 (0.78~1.34)	0.891
MECPP	5.39 (3.53~8.25)	<0.001	5.20 (3.22~8.41)	<0.001

Adjusting factors: Maternal age, educational attainment, history of miscarriage, number of previous pregnancies, pre-pregnancy body mass index (BMI), occupational history, husband’s educational attainment, husband’s employment history, maternal smoking status, creatinine and husband’s smoking status. Using univariate and multivariate logistic regression analyses were employed, with *p* < 0.05 considered statistically significant. MMP (mono-methyl), MEP (mono-methyl phthalate) MIBP (mono-ethyl phthalate), MBP (mono-butyl phthalate), MOP (mono-octyl phthalate), MBZP (mono-benzyl phthalate), MEHP (mono(2-ethylhexyl) phthalate), MEOHP (mono(2-ethyl-5-oxohexyl) phthalate), MEHHP (mono(2-ethyl-5-hydroxyhexyl) phthalate), MECPP (mono-5-carboxy-2-ethylpentyl ester).

**Table 4:** Association between PAE metabolites quantiles and spontaneous abortion.

Quantile	Univariate		Multivariate	
	OR (95%CI)	*FDR-Adjusted p-Value*	OR (95%CI)	*FDR-Adjusted p-Value*
MMP quantile				
Q1	1.00 (Reference)		1.00 (Reference)	
Q2	4.42 (2.06~9.48)	<0.001	6.20 (2.39~16.08)	<0.001
Q3	5.62 (2.64~11.95)	<0.001	8.87 (3.37~23.30)	<0.001
Q4	2.77 (1.26~6.11)	0.008	2.67 (0.94~7.60)	0.054
MEP quantile				
Q1	1.00 (Reference)		1.00 (Reference)	
Q2	2.08 (1.11~3.89)	0.018	0.85 (0.36~2.04)	0.699
Q3	1.25 (0.65~2.43)	0.486	0.56 (0.22~1.42)	0.196
Q4	1.60 (0.84~3.04)	0.125	0.68 (0.24~1.92)	0.443
MIBP quantile				
Q1	1.00 (Reference)		1.00 (Reference)	
Q2	1.10 (0.59~2.05)	0.732	0.86 (0.34~2.17)	0.698
Q3	1.10 (0.59~2.05)	0.712	0.73 (0.25~2.14)	0.516
Q4	1.21 (0.66~2.24)	0.516	0.66 (0.20~2.24)	0.467
MBP quantile				
Q1	1.00 (Reference)		1.00 (Reference)	
Q2	1.00 (0.54~1.84)	0.978	0.46 (0.18~1.19)	0.103
Q3	1.05 (0.57~1.92)	0.843	0.56 (0.21~1.48)	0.203
Q4	0.95 (0.51~1.76)	0.845	0.37 (0.12~1.17)	0.078
MEHP quantile				
Q1	1.00 (Reference)		1.00 (Reference)	
Q2	1.93 (1.01~3.71)	0.035	1.38 (0.58~3.29)	0.435
Q3	1.20 (0.60~2.40)	0.534	0.72 (0.28~1.81)	0.449
Q4	2.74 (1.45~5.16)	0.0015	1.56 (0.61~4.00)	0.299
MOP quantile				
Q1	1.00 (Reference)		1.00 (Reference)	
Q2	2.04 (1.05~3.95)	0.029	2.44 (0.95~6.29)	0.058
Q3	1.36 (0.68~2.73)	0.316	1.38 (0.49~3.91)	0.502
Q4	2.97 (1.56~5.65)	<0.001	6.02 (1.98~18.28)	0.0016
MBZP quantile				
Q1	1.00 (Reference)		1.00 (Reference)	
Q2	1.56 (0.84~2.91)	0.115	1.18 (0.48~2.90)	0.685
Q3	1.17 (0.62~2.23)	0.587	0.75 (0.27~2.07)	0.516
Q4	1.49 (0.80~2.79)	0.186	0.39 (0.13~1.16)	0.085
MEOHP quantile				
Q1	1.00 (Reference)		1.00 (Reference)	
Q2	6.58 (2.19~19.77)	<0.001	15.72 (4.41~56.05)	<0.001
Q3	16.65 (5.73~48.42)	<0.001	48.77 (13.30~178.87)	<0.001
Q4	14.30 (4.90~41.69)	<0.001	49.74 (13.14~188.26)	<0.001
MEHHP quantile				
Q1	1.00 (Reference)		1.00 (Reference)	
Q2	1.63 (0.88~3.03)	0.096	1.15 (0.49~2.70)	0.698
Q3	1.06 (0.55~2.03)	0.832	0.81 (0.35~1.89)	0.587
Q4	1.56 (0.84~2.91)	0.143	0.70 (0.36~1.58)	0.476
MECPP quantile				
Q1	1.00 (Reference)		1.00 (Reference)	
Q2	2.07 (1.00~4.31)	0.050	2.21 (0.91~5.37)	0.074
Q3	1.27 (0.58~2.77)	0.513	2.06 (0.81~5.21)	0.102
Q4	7.24 (3.65~14.36)	<0.001	12.26 (4.90~30.66)	<0.001

Adjusting factors: Maternal age, educational attainment, history of miscarriage, number of previous pregnancies, pre-pregnancy body mass index (BMI), occupational history, husband’s educational attainment, husband’s employment history, maternal smoking status, creatinine and husband’s smoking status. Using quantile univariate and multivariate logistic regression analyses, *p* < 0.05 was considered statistically significant. MMP (mono-methyl), MEP (mono-methyl phthalate) MIBP (mono-ethyl phthalate), MBP (mono-butyl phthalate), MOP (mono-octyl phthalate), MBZP (mono-benzyl phthalate), MEHP (mono(2-ethylhexyl) phthalate), MEOHP (mono(2-ethyl-5-oxohexyl) phthalate), MEHHP (mono(2-ethyl-5-hydroxyhexyl) phthalate), MECPP (mono-5-carboxy-2-ethylpentyl ester).

## Data Availability

The original contributions presented in this study are included in the article/Supplementary Materials. Further inquiries can be directed to the corresponding author.

## Supplementary Materials

The following supporting information can be downloaded at https://www.mdpi.com/article/10.3390/toxics14040300/s1, Table S1: On-board specific information table for PAE metabolites; Table S2: Limit of detection (LOD), limit of quantification (LOQ), recovery rate, and precision of metabolism of PAE; Table S3: Association between PAE metabolites and spontaneous abortion (before creatinine correction); Table S4: Association between PAE metabolites quantiles and spontaneous abortion (before creatinine correction).
