# Supplementary material for: Association Between Urinary Phthalate Metabolites and Early Spontaneous Abortion
Source: Toxics. 2026 Mar 30;14(4):300. doi: 10.3390/toxics14040300 (PMC13120065; doi:10.3390/toxics14040300)
Supplement: Supplementary file 1 [file toxics-14-00300-s001.zip › toxics-4155502-supplementary.pdf]

Table S1 On-board specific information table for phthalate metabolites

metabolite	retention time (min)	parent ion	daughter ion	collision energy
MMP	16.731	237	89	15
MEP	17.837	251	75	15
MIBP	17.840	223	75	15
MBP	19.785	223	75	15
MOP	20.426	223	73	15
MEHP	21.669	221	73	15
MEOHP	24.633	221	73	15
MBZP	27.261	179	105	20
MEHHP	29.367	221	73	15
MECPP	31.667	221	73	15

MMP (Mono-methyl), MEP (Mono-methyl phthalate) MiBP (Mono-ethyl phthalate), MBP (Mono-butyl phthalate), MOP (Mono-octyl phthalate), MBzP (Mono-benzyl phthalate), MEHP (Mono(2-ethylhexyl) phthalate), MEOHP (Mono(2-ethyl-5-oxohexyl) phthalate), MEHHP (Mono(2-ethyl-5-hydroxyhexyl) phthalate), MECPP (mono-5-carboxy-2- ethylpentyl ester).

Table S2 Limit of detection (LOD), limit of quantification (LOQ), recovery rate, and precision of metabolism of PAE.

compound	internal standard	regression equation	Decision coefficient (R^2)	LOD (μg/L Cr)	LOQ (μg/L Cr)	recovery rate （%）	precision （%）
MEP	MEHP-C4	Y=0.9972x+0.0202	0.9972	0.0288	0.0962	102.31	6.15
MMP	MEHP-C4	y=0.0037x+5.9620	0.9986	0.0375	0.1250	80.45	8.37
MIBP	MEHP-C4	Y=0.1002x+0.0813	0.9988	0.0023	0.0075	99.71	4.95
MBP	MEHP-C4	Y=0.1681x+0.1936	0.9982	0.0015	0.0051	92.64	4.77
MOP	MEHP-C4	Y=0.1300x+0.0021	0.9997	0.0625	0.2083	100.00	3.15
MEHP	MEHP-C4	Y=0.1090x+0.0234	0.9999	0.0326	0.1087	112.59	3.59
MEOHP	MEHHP-C4	Y=0.0834x-0.0876	0.9986	1.0714	3.5714	105.16	4.14
MBZP	MEHP-C4	Y=0.0877x+0.0351	0.9976	0.0405	0.1351	92.01	5.83
MEHHP	MEHHP-C4	y=0.2029x-0.0621	0.9998	0.0103	0.1087	104.21	3.67
MECPP	MEHHP-C4	Y=0.0061x-0.0070	0.9847	2.4375	3.125	124.28	5.40

R^2: Regression coefficient. Cr: creatinine. MMP (Mono-methyl), MEP (Mono-methyl phthalate) MiBP (Mono-ethyl phthalate), MBP (Mono-butyl phthalate), MOP (Mono-octyl phthalate), MBzP (Mono-benzyl phthalate), MEHP (Mono(2-ethylhexyl) phthalate), MEOHP (Mono(2-ethyl-5-oxohexyl) phthalate), MEHHP (Mono(2-ethyl-5-hydroxyhexyl) phthalate), MECPP (mono-5-carboxy-2- ethylpentyl ester).

Table S3 Association between PAE metabolites and spontaneous abortion (before creatinine correction)

Variables	Univariate		Multivariate	
	OR (95%CI)	*FDR-adjusted p-value*	OR (95%CI)	*FDR-adjusted p-value*
MMP	1.35 (1.12 ~ 1.98)	**<.001**	1.51 (1.03 ~ 2.21)	**0.009**
MEP	1.45 (1.04 ~ 2.02)	**0.012**	0.92 (0.51 ~ 1.57)	0.721
MIBP	1.12 (0.76 ~ 1.61)	0.417	0.67 (0.28 ~ 1.42)	0.243
MBP	1.13 (0.71 ~ 1.71)	0.443	0.50 (0.21 ~ 1.12)	0.053
MEHP	1.61 (1.22 ~ 2.08)	**<.001**	1.21(0.80 ~ 1.76)	0.154
MOP	1.73 (1.24 ~ 2.45)	**<.001**	1.97 (1.10 ~ 3.42)	**0.004**
MBZP	1.22 (0.87 ~ 1.64)	0.085	1.38 (0.71 ~ 1.68)	0.101
MEOHP	2.60 (1.86 ~ 3.45)	**<.001**	2.81 (1.78 ~ 4.09)	**<.001**
MEHHP	1.37 (1.08 ~ 1.73)	**0.002**	1.01 (0.75 ~ 1.30)	0.872
MECPP	5.34 (3.50 ~ 8.13)	**<.001**	5.13 (3.17 ~ 8.34)	**<.001**

Adjusting factors: maternal age, educational attainment, history of miscarriage, number of previous pregnancies, pre-pregnancy body mass index (BMI), occupational history, husband's educational attainment, husband's employment history, maternal smoking status, and husband's smoking status. Using univariate and multivariate logistic regression analyses were employed, with $p < 0.05$ considered statistically significant. MMP (Mono-methyl), MEP (Mono-methyl phthalate) MiBP (Mono-ethyl phthalate), MBP (Mono-butyl phthalate), MOP (Mono-octyl phthalate), MBzP (Mono-benzyl phthalate), MEHP (Mono(2-ethylhexyl) phthalate), MEOHP (Mono(2-ethyl-5-oxohexyl) phthalate), MEHHP (Mono(2-ethyl-5-hydroxyhexyl) phthalate), MECPP (mono-5-carboxy-2-ethylpentyl ester).

Table S4 Association between PAE metabolites quantiles and spontaneous abortion (before creatinine correction)

Quantile	Univariate		Multivariate	
	OR (95%CI)	*FDR-adjusted p-value*	OR (95%CI)	*FDR-adjusted p-value*
MMP quantile				
Q1	1.00 (Reference)		1.00 (Reference)	
Q2	4.34 (2.02 ~ 9.31)	**<.001**	6.12 (2.28 ~ 15.76)	**<.001**
Q3	5.53(2.60 ~ 11.61)	**<.001**	8.24 (3.19 ~ 21.45)	**<.001**
Q4	2.68 (1.21 ~ 6.02)	**0.007**	2.49 (0.87 ~ 7.12)	0.053
MEP quantile				
Q1	1.00 (Reference)		1.00 (Reference)	
Q2	2.01 (1.07 ~ 3.82)	**0.016**	0.79 (0.31 ~ 1.89)	0.615
Q3	1.22 (0.61 ~ 2.39)	0.473	0.52 (0.21 ~ 1.37)	0.187
Q4	1.57 (0.81 ~ 3.01)	0.121	0.60 (0.21 ~ 1.85)	0.421
MIBP quantile				
Q1	1.00 (Reference)		1.00 (Reference)	
Q2	1.07 (0.56 ~ 2.01)	0.703	0.82 (0.31 ~ 2.08)	0.635
Q3	1.08 (0.57 ~ 2.03)	0.701	0.71 (0.22 ~ 2.07)	0.502
Q4	1.18 (0.62 ~ 2.22)	0.503	0.62 (0.17 ~ 2.08)	0.426
MBP quantile				
Q1	1.00 (Reference)		1.00 (Reference)	
Q2	1.00 (0.52 ~ 1.81)	0.945	0.42 (0.16 ~ 1.07)	0.092
Q3	1.02 (0.53 ~ 1.87)	0.821	0.52 (0.21 ~ 1.43)	0.201
Q4	0.91 (0.47 ~ 1.72)	0.812	0.31 (0.11 ~ 1.14)	0.072
MEHP quantile				
Q1	1.00 (Reference)		1.00 (Reference)	
Q2	1.90 (1.01 ~ 3.65)	**0.031**	1.32 (0.55 ~ 3.21)	0.412
Q3	1.16 (0.57 ~ 2.37)	0.509	0.69 (0.22 ~ 1.76)	0.417
Q4	2.69 (1.41 ~ 5.09)	**0.0012**	1.52 (0.54 ~ 3.79)	0.253
MOP quantile				
Q1	1.00 (Reference)		1.00 (Reference)	
Q2	2.01 (1.03 ~ 3.84)	**0.026**	2.41 (0.90 ~ 6.17)	0.055
Q3	1.33 (0.61 ~ 2.67)	0.307	1.32 (0.43 ~ 3.79)	0.467

Q4	2.92 (1.51 ~ 5.63)	**<.001**	5.79 (1.92 ~ 16.79)	**0.0012**
MBZP quantile				
Q1	1.00 (Reference)		1.00 (Reference)	
Q2	1.52 (0.80 ~ 2.87)	0.112	1.12 (0.41 ~ 2.76)	0.619
Q3	1.13 (0.58 ~ 2.17)	0.532	0.71 (0.22 ~ 2.01)	0.501
Q4	1.45 (0.76 ~ 2.71)	0.182	0.32(0.11 ~ 1.14)	0.079
MEOHP quantile				
Q1	1.00 (Reference)		1.00 (Reference)	
Q2	6.51 (2.13 ~ 18.67)	**<.001**	15.64 (4.28 ~ 52.38)	**<.001**
Q3	16.32 (5.46 ~ 44.37)	**<.001**	45.73 (11.28 ~ 169.34)	**<.001**
Q4	14.19 (4.64 ~ 39.43)	**<.001**	45.12 (11.34 ~ 176.26)	**<.001**
MEHHP quantile				
Q1	1.00 (Reference)		1.00 (Reference)	
Q2	1.57 (0.82 ~ 2.95)	0.087	1.12 (0.45~ 2.59)	0.654
Q3	1.02 (0.50 ~ 2.00)	0.812	0.73 (0.31 ~ 1.82)	0.557
Q4	1.52 (0.81 ~ 2.79)	0.125	0.62 (0.33 ~ 1.54)	0.457
MECPP quantile				
Q1	1.00 (Reference)		1.00 (Reference)	
Q2	2.02 (0.98 ~ 4.27)	0.054	2.12 (0.79 ~ 4.97)	0.069
Q3	1.21 (0.52 ~ 2.73)	0.498	2.01 (0.76 ~ 4.69)	0.098
Q4	7.13 (3.49 ~ 13.24)	**<.001**	12.09 (4.35 ~ 28.97)	**<.001**

Adjusting factors: maternal age, educational attainment, history of miscarriage, number of previous pregnancies, pre-pregnancy body mass index (BMI), occupational history, husband's educational attainment, husband's employment history, maternal smoking status, and husband's smoking status.Using quantile univariate and multivariate logistic regression analyses, $p < 0.05$ was considered statistically significant. MMP (Mono-methyl), MEP (Mono-methyl phthalate) MiBP (Mono-ethyl phthalate), MBP (Mono-butyl phthalate), MOP (Mono-octyl phthalate), MBzP (Mono-benzyl phthalate), MEHP (Mono(2-ethylhexyl) phthalate), MEOHP (Mono(2-ethyl-5-oxohexyl) phthalate), MEHHP (Mono(2-ethyl-5-hydroxyhexyl) phthalate), MECPP (mono-5-carboxy-2-ethylpentyl ester).
